# Information sources for obesity prevention policy research: a review of systematic reviews

**DOI:** 10.1186/s13643-017-0543-2

**Published:** 2017-08-08

**Authors:** Rosie Hanneke, Sabrina K. Young

**Affiliations:** 1 Library of the Health Sciences–Chicago, University of Illinois at Chicago, 1750 W. Polk St, Chicago, IL 60612 USA; 2 Health Policy and Administration, School of Public Health, University of Illinois at Chicago, 1603 W. Taylor St, Chicago, IL 60612 USA; 3 The Cancer Education and Cancer Development Program, Institute for Health Research and Policy, 1747 W. Taylor St, Chicago, IL 60608 USA

**Keywords:** Review literature as topic, Information storage and retrieval, Bibliographic databases, Systematic review methodology, Obesity prevention, Health policy

## Abstract

**Background:**

Systematic identification of evidence in health policy can be time-consuming and challenging. This study examines three questions pertaining to systematic reviews on obesity prevention policy, in order to identify the most efficient search methods: (1) What percentage of the primary studies selected for inclusion in the reviews originated in scholarly as opposed to gray literature? (2) How much of the primary scholarly literature in this topic area is indexed in PubMed/MEDLINE? (3) Which databases index the greatest number of primary studies not indexed in PubMed, and are these databases searched consistently across systematic reviews?

**Methods:**

We identified systematic reviews on obesity prevention policy and explored their search methods and citations. We determined the percentage of scholarly vs. gray literature cited, the most frequently cited journals, and whether each primary study was indexed in PubMed. We searched 21 databases for all primary study articles not indexed in PubMed to determine which database(s) indexed the highest number of these relevant articles.

**Results:**

In total, 21 systematic reviews were identified. Ten of the 21 systematic reviews reported searching gray literature, and 12 reviews ultimately included gray literature in their analyses. Scholarly articles accounted for 577 of the 649 total primary study papers. Of these, 495 (76%) were indexed in PubMed. Google Scholar retrieved the highest number of the remaining 82 non-PubMed scholarly articles, followed by Scopus and EconLit. The *Journal of the American Dietetic Association* was the most-cited journal.

**Conclusions:**

Researchers can maximize search efficiency by searching a small yet targeted selection of both scholarly and gray literature resources. A highly sensitive search of PubMed and those databases that index the greatest number of relevant articles not indexed in PubMed, namely multidisciplinary and economics databases, could save considerable time and effort. When combined with a gray literature search and additional search methods, including cited reference searching and consulting with experts, this approach could help maintain broad retrieval of relevant studies while improving search efficiency. Findings also have implications for designing specialized databases for public health research.

**Electronic supplementary material:**

The online version of this article (doi:10.1186/s13643-017-0543-2) contains supplementary material, which is available to authorized users.

## Background

Published systematic reviews on obesity prevention policy synthesize evidence from a diverse collection of resources, including scholarly and gray literature from disciplines ranging from medicine and nutrition to political science and education. These systematic reviews, largely produced by academic researchers [1], aim to capture the array of scientific literature available for a topic and are a valuable tool for evidence-based policy and practice [2]. Systematic identification of evidence in the public health setting can be challenging in a number of ways; among other challenges, given the interdisciplinary nature of the field [3], literature on a single topic may not be concentrated in one location or described using uniform terminology. It is necessary, therefore, to identify search methods that are both effective and efficient, and which allow for the creation of comprehensive systematic reviews to inform policymaking.

The present study is the first to assess citation patterns of reviews on obesity prevention policies—a topic growing in relevance as growing obesity levels in the USA [4, 5] become a national concern. Previous studies from the UK that have analyzed the citation patterns of systematic reviews in other public health topics found that PubMed [6] and MEDLINE [7] are common sources in which to find articles. Levay, Raynor, and Tuvey [7] examined the publications included in three systematic reviews which informed National Institute for Health and Care Excellence (NICE) Public Health Guidance. The three reviews focused on obesity, spatial planning, and tuberculosis. In addition to their MEDLINE analysis, the authors also found that literature resources outside of database searching, including citation lists and websites, contributed 42% of the resources used by NICE. A similar analysis of a systematic review in occupational injury indicated that multiple information sources should be searched in order to identify all relevant literature, including but not limited to the peer-reviewed journals indexed in scholarly databases, as much of the relevant evidence is published outside of the scholarly literature [8].

This study examines three research questions pertaining to systematic reviews on nutrition and physical activity policy: (1) What percentage of the primary studies selected for inclusion in the reviews originated in scholarly as opposed to gray literature? (2) How much of the primary scholarly literature in this topic area is indexed in PubMed/MEDLINE? (3) Which databases index the greatest number of primary studies not indexed in PubMed, and are these databases searched consistently across systematic reviews—or were they only searched in a smaller subset of the reviews? Consistent with previous literature, we hypothesized that the majority of articles cited would be scholarly journal articles indexed in PubMed. We also hoped to identify lesser-used databases and individual journals that could contribute to more comprehensive, efficient systematic searches.

## Methods

### Research strategy

The three research questions all serve to address the following central question: What is the potential benefit, in terms of additional relevant studies identified, for authors of a systematic review to search a given database in addition to PubMed? It is not possible to answer this question directly using just the citations included in systematic reviews, since a single article included in a review may be indexed in multiple databases. We therefore designed our research strategy around answering the three questions described above, which serve as proxies to indirectly answer our central research question.

### Data sources

We identified potential systematic reviews through searches of the following databases, without publication date restrictions: PubMed, Worldwide Political Science Abstracts, Public Affairs Information Service (PAIS) International, Scopus, and Web of Science. We ran test searches on multiple databases, chosen based on author expertise and on the search strategies of other reviews in health policy, before these five were ultimately selected for our search. The search strategy [see Additional file 1] consisted of controlled vocabulary terms and keywords representing the two conceptual domains of policy (*policy*, *legislation*, etc.) and obesity prevention (obes*, *physical activity*, etc.), combined with keywords designed to identify systematic review methodology, including *systematic* and meta*.

### Study selection

The two study authors separately screened titles and abstracts of the search results, blinded to author and journal, for potential inclusion, resolving any disagreements through discussion. To qualify for inclusion, the following criteria were required: English language systematic review evaluating the effectiveness of an obesity prevention policy or set of policies, including or exclusively focusing on US-based policy. Obesity prevention policies include laws and regulations supporting healthy behaviors in nutrition and/or physical activity, with policy defined as an action taken by a federal, state, or local governmental body. Actions taken by governmental bodies are universally enforceable and therefore relevant for a variety of researchers with an interest in policy. Examples of obesity prevention policies include sugar-sweetened beverage taxes (often termed “soda taxes”), school food and beverage regulations, and complete streets policies which require space for all types of users (including cyclists and pedestrians) on the road. Systematic reviews on obesity prevention interventions—such as specific physical education teaching strategies—were not included.

We determined whether a review was “systematic” by examining the methods, which had to be explicitly described. If review authors described their search in sufficient detail to identify the databases searched, and provided a clear numerical description of results (e.g., in a PRISMA flow diagram [9]), the article was included. We determined that all included reviews shared the aims outlined in the Cochrane Handbook, which states that systematic reviews “seek to collate all evidence that fits pre-specified eligibility criteria in order to address a specific research question,” and “aim to minimize bias by using explicit, systematic methods” [10]. Beyond this broad assessment of each review’s aims and search methodology, we did not assess the quality of reviews, nor did we exclude reviews on the basis of quality.

### Data extraction

From each review, we extracted the review objective and findings, metadata such as publication date and author names, search methodology, and the individual citations of each primary study cited within the review. We then aggregated the primary study data to determine the percentages of scholarly and gray literature cited, the most frequently cited journals, and whether each study was indexed in the PubMed database. When a primary study was cited by more than one review, the duplicate citations were not deleted but were analyzed as additional data. We defined “gray literature” as any study included in a systematic review that did not originate in traditional publishing venues, namely, in scholarly journals. This included dissertations, conference proceedings, and reports from government and other organizations. PubMed searches the MEDLINE collection of health sciences citations and abstracts as well as additional journals not indexed in MEDLINE [11]. We chose to search for articles in PubMed rather than searching MEDLINE through a subscription-based platform (e.g., Ovid or EBSCOhost) due to the fact that, as a freely available resource, it is most likely to be searched by policymakers and other researchers not affiliated with an academic institution.

We compiled a list of all the scholarly databases that review authors reported searching, a total of 30. We searched these databases for all primary study articles not indexed in PubMed, in order to determine which database(s) indexed the highest number of these relevant articles. From the list of 30 databases, we searched PubMed first. All articles in MEDLINE would also be in PubMed. This left 28 databases. Seven of these databases were not licensed through our institution’s library so we were unable to access them: British Education Index, CAB Abstracts, Cambridge Science Abstracts, CSA Environmental Sciences, Geobase, Global Health, and SportDiscus. The remaining 21 databases include AgEcon, Australian Education Index, Business Source Premier, CINAHL (we searched CINAHL Plus with Full Text), Cochrane Library, EconLit, Embase, ERIC, Global Health Library, Google Scholar, LILACS, PAIS, PsycINFO, Science Citation Index, ScienceDirect, Scopus, Social Sciences Citation Index, Sociological Abstracts, TRIS online (we searched TRID), Web of Science, and Web of Knowledge. We searched these 21 databases for the non-PubMed articles by title, author, or a combination of those elements, until we could confidently determine whether each article was indexed in each database.

## Results

**Fig. 1:**
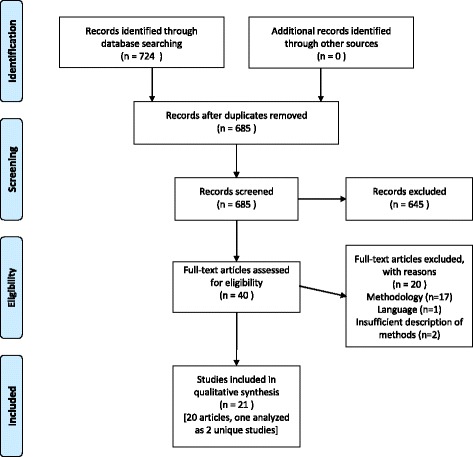
Review flow diagram

From our initial search for systematic reviews, we exported 724 resulting citations to citation management software, after which 39 duplicate records were deleted, leaving 685 unique citations. We reviewed the full text of 40 articles that potentially met our criteria. Of these, 17 were excluded for reasons relating to their methodology (not systematic) or objective (e.g., review of policy content rather than effectiveness). One review was excluded at this stage because of language (Spanish), and two reviews were ineligible for analysis because of insufficient information provided on search methodology. The final set of articles, therefore, equaled 20 systematic reviews. In one of these articles [12], authors conducted a review of two distinct topics—(1) the price elasticity of demand for sugar-sweetened beverages, fast food, and fruits and vegetables and (2) associations of food and beverage prices and/or taxes with body weight. Because the types of primary studies used for these two inquiries are sufficiently different, we considered these two analyses separately, for a total of 21 systematic review studies within 20 papers (refs. [12–31]) (Fig. 1). A table containing further details about each review, including primary objective and findings, is available as a supplement to this paper [Additional file 2].

### Search methods

The 21 systematic reviews represented a broad range in the number and type of information sources they reported searching. All 21 reviews searched PubMed and/or Ovid MEDLINE, the only universally searched database. Of the 21 reviews, 10 reported searching at least one gray literature resource. In addition to searching for scholarly and gray literature in traditional bibliographic databases or on the web, complementary search methods included the following: Four of the reviews reported that experts contributed to the literature search process, although this process was not described transparently. None of the reviews reported forward citation searching (studies that have cited those included in the review). Several reported backward citation searching, including searching reference lists of included studies (*n* = 7) and relevant review articles (*n* = 6). In two reviews [18, 30], it was reported that reference lists were scanned for relevant studies, but it was unclear whether this referred to the reference lists of included studies, review articles, both, or another source. Finally, one review [17] handsearched an individual journal archive, and one [30] reported using the “related articles” feature in PubMed to identify additional studies.

The number of resources searched by review authors ranged from 1 [23] to 18 [13]. The median number of resources searched was 5 (±4.23). This number included both scholarly and gray literature databases, websites, and search engines. It does not include complementary search methods, such as articles identified through reference lists or by subject experts. When gray literature search strategies were reported as “various” or simply “gray literature,” we counted them as a single resource. When multiple gray literature resources were explicitly mentioned by name, we counted these individually.

### Number of sources cited

The systematic reviews varied widely in the total number of papers they included in their respective analyses (*M* = 30.86, SD ± 21.38, range = 7–94). This reflects the number of publications, not studies, since multiple publications can result from a single study. Similarly, it is possible that multiple studies could have been condensed into a single publication. Ten reviews reported both the number of papers included and the number of unique studies represented by these papers. Three of the 10 reported the same number for both categories, indicating no multiple publications from any of the included studies. The remaining 11 reviews reported only one number.

### Scholarly literature

Scholarly articles accounted for 577 of the 649 total primary study papers cited in the systematic reviews. The majority of citations within both the aggregated 649 primary studies and within each individual review were from scholarly literature, with the exception of one review [16] which focused on transportation, cycling, and walking. In this review, 13 gray and 3 scholarly sources were cited.

Of the 21 reviews, 11 searched for scholarly articles exclusively. Review authors reported searching for scholarly articles in 30 unique bibliographic databases; the five most-searched were PubMed and/or MEDLINE (*n* = 21), CINAHL (*n* = 10), EconLit (*n* = 8), Cochrane Library (*n* = 8), and Public Affairs Information Service (*n* = 6). Eleven reviews searched MEDLINE through PubMed, and one review searched MEDLINE through Ovid. Five reviews reported searching both PubMed and Ovid MEDLINE, and the remaining four reported MEDLINE in their search strategy without specifying a platform. Several search strategies included databases within the ISI Web of Science platform/family (which has a complicated naming history), including Web of Science (*n* = 5), Web of Knowledge (*n* = 4) (the former name of the platform), Social Sciences Citation Index (*n* = 1), and Science Citation Index (*n* = 1).

### Most-cited scholarly journals

The 577 scholarly journal articles originated in 151 unique journals, 85 of which were cited only once. The *Journal of the American Dietetic Association* was the most-cited journal overall, cited 38 times within the 21 reviews (see Table 1). The number of unique journals cited within a single systematic review varied (*M* = 16, SD ± 10.74, range = 2–48). When tabulated within individual systematic reviews, *Health Affairs* was the top journal for the most reviews; it was the most-cited journal in five reviews. The *American Journal of Public Health* was cited in more systematic reviews (*n* = 13) than any other journal.

**Table 1:** Summary of the 20 most-cited journals in 21 systematic reviews

Journal name	Times cited	Cited in *n* reviews
J Am Diet Assoc	38	12
Am J Public Health	32	13
Public Health Nutr	25	10
Am J Prev Med	24	9
Prev Med	19	9
Health Aff (Millwood)	18	9
J Nutr	14	9
J Sch Health	14	8
Am J Agric Econ	12	7
Arch Intern Med	12	6
J Nutr Educ Behav	12	5
Prev Chronic Dis	12	5
Health Econ	11	8
J Health Econ	11	7
Am J Clin Nutr	10	6
Health Place	10	6
Contemp Econ Policy	9	8
Econ Hum Biol	9	8
Health Promot Pract	9	5
J Adolesc Health	9	7

### Indexing in PubMed vs. other databases

Of all 577 scholarly articles included in the 21 reviews, 495 (86%) were included in PubMed as of June 2016, i.e., the articles were indexed in PubMed, although review authors may have identified them elsewhere. Of the remaining 82 scholarly articles not in PubMed, the greatest number was retrieved with Google Scholar, followed by Scopus and EconLit (see Table 2). The Google Scholar search retrieved all 82 articles, Scopus retrieved 66, and EconLit retrieved 61.

**Table 2:** Database indexing of scholarly articles included in 21 systematic reviews, alone and with PubMed

Database	Non-PubMed scholarly articles retrieved (of 82)	Total articles retrieved (of 577) when searched in combination with PubMed	Percentage of total articles retrieved (of 577) when searched in combination with PubMed	Number of systematic reviews that searched this database
Google Scholar	82	577	100%	3
Scopus	66	561	97.23%	1
EconLit	61	556	96.36%	8
Web of Knowledge (Web of Science All Databases)	58	553	95.84%	4
Web of Science (Web of Science core collection)	58	553	95.84%	5
Social Sciences Citation Index	52	547	94.80%	1
Business Source Premier	46	541	93.76%	1
Science Citation Index	31	526	91.16%	1
ScienceDirect (platform, full-text content may vary by institutional subscription)	21	516	89.43%	1
PAIS	13	508	88.04%	6
AgEcon	6	501	86.83%	1
Embase	6	501	86.83%	3
CINAHL (plus with full text)	4	499	86.48%	10
TRIS online (searched TRID)	3	498	85.79%	1
PsycINFO	2	497	86.14%	5
ERIC	1	496	85.96%	3
Sociological Abstracts	1	496	85.96%	1
Australian Education Index	0	495	85.79%	1
Cochrane Library (includes CENTRAL and Cochrane Database of Systematic Reviews)	0	495	85.79%	8
Global Health Library	0	495	85.79%	1
LILACS	0	495	85.79%	1

### Gray literature

While it is possible to discover gray literature through certain bibliographic databases, such as Public Affairs Information Service (PAIS), EconLit, and Business Source Premier, 10 reviews reported searching at least one additional gray literature resource. Some reviews named specific resources, while in others, it was simply reported that “gray literature” or “websites” were searched. Two reviews stated that gray literature was searched without specifying a particular database, website, or search engine; four reviews mentioned searching Google for non-scholarly papers; others named individual gray literature resources within the list of databases searched. These included clinical trial registries such as ClinicalTrials.gov, dedicated gray literature databases such as OpenGrey.eu, organizational websites such as the World Health Organization and the National Bureau of Economic Research, and the websites of national school lunch programs.

Gray literature was ultimately included in 12 reviews, including 9 of the 10 reviews that reported searching gray literature. An additional three reviews included gray literature but did not indicate that gray literature resources were searched; these references may have been identified from a bibliography, by an expert, or using another method of discovery. Among these 12 reviews, the number of included studies from gray sources ranged from 1 to 13 (*M* = 6, SD ± 4.43).

## Discussion

### Summary of major findings

The results of this study indicate that PubMed is the most fruitful source of scholarly literature relevant to systematic reviews of obesity prevention policy. The databases that index the greatest number of relevant articles not included in PubMed are the large multidisciplinary databases Google Scholar, Scopus, and Web of Science; and databases specializing in economics, namely, EconLit and Business Source Premier. EconLit and Business Source Premier appear especially useful for reviews of economic interventions such as food and beverage taxes or subsidies. Larger databases like Google Scholar, Scopus, and Web of Science cover an expansive body of literature across many disciplines; however, this means that searching these resources may produce unwieldy search results, with potentially large numbers of irrelevant citations.

### Scholarly literature searches

When developing search strategies, review authors are not necessarily selecting databases in a manner that maximizes search retrieval. For example, 10 systematic reviews searched CINAHL, making it the second most-searched resource. However, CINAHL only indexed 4 of the 82 relevant articles not indexed in PubMed. By contrast, Scopus, which indexed 66 of the 82 articles, was searched in only 1 review. This implies that the majority of relevant articles identified through CINAHL searches could also be located with a comprehensive search of PubMed. It should be noted that if such a PubMed search were not adequately comprehensive, i.e., if it were not a highly sensitive search, it would be problematic to assume that all relevant articles would be retrieved. This has implications not only for the question of whether to search additional databases, but also for how systematic review searching is approached more broadly speaking. When designing the search methodology for a systematic review, researchers must decide for themselves which balance of specificity vs. sensitivity best suits the aims and parameters of their project.

The fact that searches of Google Scholar retrieved all non-PubMed articles implies that one could potentially locate all relevant scholarly literature by searching these two databases. However, the two processes of searching for a known item by title and discovering that same article within a large list of results when searching a database with keywords are profoundly different [32]. This fact applies to all databases searched in the present study. We assessed each database’s ability to retrieve relevant studies by searching for those studies by title; it is entirely possible that a keyword search of the same database may not retrieve the same studies, especially if the search strategy were not sufficiently broad.

Google Scholar in particular has been found to be problematic when searched for systematic reviews, for a few reasons. These range from the inability to create a structured and reproducible search strategy to the limits Google Scholar places on search syntax and viewing results [33, 34]. While all relevant non-PubMed primary studies were indexed by Google Scholar, they may not be retrievable by keyword search in Google Scholar given these limitations. Therefore, it would be imprudent to assume that all relevant literature could be identified through Google Scholar alone in a systematic review. However, it may prove useful in identifying gray and scholarly literature beyond that which can be identified using traditional search methods [35, 36] and should therefore be considered as one of multiple resources to search.

### Gray literature searches

Of the 10 reviews that reported searching gray resources, all but one ultimately included gray literature of some kind in their analyses. An additional three reviews that did not report searching gray resources nonetheless included relevant gray literature in their analyses. Similar to what has been found elsewhere [37–39], this indicates that searching gray resources, e.g., organizational websites or conference proceedings, in addition to traditional bibliographic databases, may likely produce relevant data for research syntheses. Despite the additional time this requires, searching the gray literature broadens the scope of a review and helps researchers avoid potential publication bias.

Some of the challenges inherent to including gray literature can be mitigated by using a systematic method for searching and identifying evidence outside of traditional databases. For example, developing a plan for the search in advance, including the sources to search and terms to use, can help keep this process manageable within the desired timeframe [39]. Stansfield, Dickson, and Bangpan [40] propose a three-stage process that allows the flexibility necessary to adapt website searches to various research topics while preserving the systematic review principles of transparency, accountability, and reproducibility.

### Individual journals

By identifying the individual journal titles that were cited most frequently, we hope that researchers may benefit in a few ways. First, systematic review guides recommend handsearching individual journal titles in addition to conducting database searches [10, 41, 42]. This can be extremely time-consuming. By focusing on just a few journals that have been identified as rich sources of literature on obesity prevention policy, review authors can include handsearching in their review in a way that is helpful but not overly burdensome. Second, any individual researcher interested in this general topic would be well served by establishing “table of contents” alerts for these relevant journals. These alerts notify the user, usually via email, when new research is published. Finally, many libraries and other institutions that facilitate journal access face uncertain financial situations. Such organizations may find this information useful when making the decision to retain or cancel a journal subscription.

### PubMed/MEDLINE and discoverability

The ubiquity of PubMed/MEDLINE is a common theme. As the only universally searched resource among all 21 systematic reviews, it stands to reason that an article would have the greatest chance of being identified in a systematic review search if it was included in PubMed/MEDLINE. In order to ensure maximum discoverability, it would behoove authors to publish their research in journals indexed for MEDLINE. The National Library of Medicine maintains a list of all journals cited in PubMed, including all journals indexed for MEDLINE [43]. While not adequate to be used as the sole resource for a systematic review, it does appear essential as one of several resources to include.

### Limitations

These conclusions are applicable to systematic reviews of obesity prevention policy and are not necessarily generalizable to other health policy or public health topics. Systematic reviews in other health policy areas may require the use of resources other than those found to be most effective in this example, due to the fact that there may be different, discipline-specific databases more appropriate for a given topic. Further, these results pertain to research on “big P” policies, comprising actions by governmental bodies; conclusions may not transfer to studies on “small p” policies, such as organizational policies or interventions. While our search was comprehensive and included several databases, we did not search any resources that specifically focus on evidence syntheses, such as Epistemonikos, Trip, and Health Evidence. There may be additional systematic reviews of obesity prevention policy that we did not identify, either in these resources or elsewhere.

The data collected on search methods and citations were limited to what review authors provided in the articles. When possible, we contacted authors for missing or unclear information. Only 5 of the 21 reviews reported a complete, line-by-line search strategy, including all search terms and indication of what fields were searched in which database. Consequently, we were unable to assess the quality of the included reviews’ search strategies. A review with a demonstrably superior search strategy would likely produce different results from a review with a flawed search strategy, especially if a search erred in favor of specificity rather than sensitivity. Reviews with insufficiently broad search strategies may fail to identify additional relevant primary studies. If this were the case with any of the reviews included in this study, it could affect the conclusions we have drawn; without complete search strategies, it is not possible to know. The incompleteness of published search strategies in the majority of systematic reviews analyzed here underlines the need for increased scrutiny at the editorial level, in order to ensure that all published systematic reviews contain truly reproducible search strategies.

No published Cochrane reviews currently exist on this topic. Given the rigor of Cochrane review methodology, it may have strengthened or otherwise affected our analysis to have included one or more of these. While no Cochrane reviews were available for inclusion in our study, as of June 2017, several protocols for proposed Cochrane reviews of food and beverage taxation that appear potentially eligible for inclusion have been published.

We analyzed 23 of the 30 databases that review authors reported searching but were unable to access the remaining 7 databases. It is possible that these databases may include many of the articles not included in PubMed, and possibly more of them than the databases we searched. While authors reported which databases they searched, it was not possible to identify the origin of articles indexed in multiple databases. Therefore, while 76% of the scholarly articles were indexed in PubMed, we cannot say for certain that this is where the authors identified all of them. If an article is indexed, for example, in both PubMed and CINAHL, the CINAHL search may have identified this article if different keywords and controlled vocabulary were used than in the PubMed search.

We located these articles individually in PubMed; as mentioned above, this is a very different process than searching PubMed using keywords. As such, searching additional databases with significant MEDLINE overlap, such as CINAHL, PsycINFO, and Embase, may be useful in that they can help “fill in the gaps” missed by a PubMed search. The data presented in Table 2 should therefore be interpreted with caution. However, these data indicate that if a highly sensitive search is run in PubMed, diminishing returns may be seen in the number of unique relevant articles added to the review by these additional databases. Some researchers may still wish to search these databases in order to ensure that nothing was missed in PubMed.

Finally, while all systematic reviews addressed policies related to obesity prevention, they were nevertheless a heterogeneous collection, including topics from transportation and physical activity to food and beverage taxes and school-based policies. Further research may include a closer analysis of the reviews that had equivalent objectives, for example, those that examined economic interventions, allowing for a more apples-to-apples comparison.

## Conclusions

As hypothesized, most studies cited in systematic reviews of obesity prevention policy come from scholarly journals and are indexed in PubMed. However, searching PubMed exclusively may exclude a substantial minority of relevant scholarly articles from the search. Google Scholar is also insufficient as a stand-alone source. Therefore, when conducting a systematic review of the literature in obesity prevention policy, an approach that includes a highly sensitive search of PubMed and a small number of additional, carefully selected databases will enable review authors to conduct a database search that is comprehensive yet limited to a manageable number of resources. Furthermore, searches for gray literature, such as government reports and working papers, often leads to the discovery of additional relevant studies. When targeted searches for scholarly and gray literature are combined with complementary search methods including cited reference searching (forwards and backwards), consulting with experts, and handsearching individual journals, broad retrieval of relevant studies can be maintained while improving search efficiency.

All search methods should be reported explicitly and transparently in the review in order to support reproducibility, a key characteristic of systematic review methodology [10] that was nonetheless absent from a majority of the reviews we assessed. This includes a line-by-line search strategy stating which database fields were searched; the names of individual gray literature resources, rather than a vague statement that “gray literature was searched”; and an explicit description of additional search methods. Reporting all search methods in detail not only enhances transparency and reproducibility of the systematic review itself but also indirectly provides guidance to other researchers looking to improve their searches on the same or similar topics.

Conclusions from this study have the potential to save academic researchers and public health practitioners considerable resources. By focusing on the databases and websites most likely to lead to relevant literature, researchers can improve search efficiency, saving both time and effort. Finally, these findings may help in the design of specialized databases for multidisciplinary public health research, as has been proposed elsewhere [44].

## Additional files

Additional file 1: Search strategy to identify systematic reviews in obesity prevention policy. This file contains the search terms used by the authors to search the databases PubMed, Public Affairs Information Service (PAIS), Worldwide Political Science Abstracts, Scopus, and Web of Science. (PDF 253 kb)

Additional file 2: Systematic reviews included in analysis. This table lists and describes the 21 systematic reviews analyzed in this paper, including metadata (review authors, title, year, journal), review objectives, and major findings. (PDF 110 kb)

## Abbreviations

Am J Agric Econ: American Journal of Agricultural Economics
Am J Clin Nutr: American Journal of Clinical Nutrition
Am J Prev Med: American Journal of Preventive Medicine
Am J Public Health: American Journal of Public Health
Arch Intern Med: Archives of Internal Medicine
CAB: Centre for Agriculture and Biosciences
CINAHL: Cumulative Index to Nursing and Allied Health Literature
Contemp Econ Policy: Contemporary Economic Policy
CSA: Cambridge Scientific Abstracts
Econ Hum Biol: Economics and Human Biology
Health Aff (Millwood): Health Affairs
Health Econ: Health Economics
Health Place: Health & Place
Health Promot Pract: Health Promotion Practice
ISI: Institute for Scientific Information
J Adolesc Health: Journal of Adolescent Health
J Am Diet Assoc: Journal of the American Dietetic Association
J Health Econ: Journal of Health Economics
J Nutr Educ Behav: Journal of Nutrition Education and Behavior
J Nutr: Journal of Nutrition
J Sch Health: Journal of School Health
LILACS: Latin American and Caribbean Health Sciences Literature
NICE: National Institute for Health and Care Excellence
PAIS: Public Affairs Information Service
Prev Chronic Dis: Preventing Chronic Disease
Prev Med: Preventive Medicine
PRISMA: Preferred Reporting Items for Systematic Reviews and Meta-Analyses
Public Health Nutr: Public Health Nutrition
TRID: Transportation Research International Documentation
TRIS: Transportation Research Information Services

## References

[1] Kite J, Indig D, Mihrshahi S, Milat A, Bauman A (2015). Assessing the usefulness of systematic reviews for policymakers in public health: a case study of overweight and obesity prevention interventions. Prev Med.

[2] Chriqui JF, Young SK, Eyler AA, Chriqui JF, Moreland-Russell S, Brownson RC (2016). Public health policy analysis and evaluation. Prevention, policy, and public health.

[3] Alpi KM (2005). Expert searching in public health. J Med Libr Assoc..

[4] Ogden CL, Caroll MD, Lawman HG, Fryar CD, Kruszon-Moran D, Kit BK, Flegal KM, Centers for Disease Control and Prevention (CDC) (2016). Trends in obesity prevalence among children and adolescents in the United States, 1988–1994 through 2013-2014. JAMA.

[5] May AL, Freedman D, Sherry B, Blanck HM (2013). Obesity—United States, 1999–2010. MMWR Suppl.

[6] Stansfield C, Brunton G, Rees R (2014). Search wide, dig deep: literature searching for qualitative research. An analysis of the publication formats and information sources used for four systematic reviews in public health. Res Synth Methods.

[7] Levay P, Raynor M, Tuvey D (2015). The contributions of MEDLINE, other bibliographic databases and various search techniques to NICE public health guidance. Evid Based Libr Inf Pract.

[8] Beahler CC, Sundheim JJ, Trapp NI (2000). Information retrieval in systematic reviews: challenges in the public health arena. Am J Prev Med.

[9] Moher D, Liberati A, Tetzlaff J, Altman DG (2009). Preferred Reporting Items for Systematic Reviews and Meta-Analyses: the PRISMA statement. Phys Ther.

[10] Higgins JPT, Green S, eds. Cochrane handbook for systematic reviews of interventions. 2011. http://handbook-5-1.cochrane.org/. Accessed 20 Apr 2017.

[11] National Library of Medicine. FAQ: PubMed®. U.S. National Library of Medicine. 2016. https://www.nlm.nih.gov/services/pubmed.html. Accessed 17 Nov 2016.

[12] Powell LM, Chriqui JF, Khan T, Wada R, Chaloupka FJ (2013). Assessing the potential effectiveness of food and beverage taxes and subsidies for improving public health: a systematic review of prices, demand and body weight outcomes. Obes Rev.

[13] Alagiyawanna A, Townsend N, Mytton O, Scarborough P, Roberts N, Rayner M (2015). Studying the consumption and health outcomes of fiscal interventions (taxes and subsidies) on food and beverages in countries of different income classifications: a systematic review. BMC Public Health.

[14] Cabrera Escobar MA, Veerman JL, Tollman SM, Bertram MY, Hofman KJ (2013). Evidence that a tax on sugar sweetened beverages reduces the obesity rate: a meta-analysis. BMC Public Health.

[15] Calancie L, Leeman J, Jilcott Pitts SB, Khan LK, Fleischhacker S, Evenson KR (2015). Nutrition-related policy and environmental strategies to prevent obesity in rural communities: a systematic review of the literature, 2002-2013. Prev Chronic Dis.

[16] Cavill N, Kahlmeier S, Rutter H, Racioppi F, Oja P (2008). Economic analyses of transport infrastructure and policies including health effects related to cycling and walking: a systematic review. Transp Policy.

[17] Chriqui JF, Pickel M, Story M (2014). Influence of school competitive food and beverage policies on obesity, consumption, and availability: a systematic review. JAMA Pediatr.

[18] Downs SM, Thow AM, Leeder SR (2013). The effectiveness of policies for reducing dietary trans fat: a systematic review of the evidence. Bull World Health Organ.

[19] Freudenberg N, Franzosa E, Sohler N, Li R, Devlin H, Albu J (2015). The state of evaluation research on food policies to reduce obesity and diabetes among adults in the United States, 2000–2011. Prev Chronic Dis.

[20] Haack SA, Byker CJ (2014). Recent population adherence to and knowledge of United States federal nutrition guides, 1992–2013: a systematic review. Nutr Rev.

[21] Jaime PC, Lock K (2009). Do school based food and nutrition policies improve diet and reduce obesity?. Prev Med.

[22] Maniadakis N, Kapaki V, Damianidi L, Kourlaba G (2013). A systematic review of the effectiveness of taxes on nonalcoholic beverages and high-in-fat foods as a means to prevent obesity trends. Clinicoecon Outcomes Res.

[23] Mayne SL, Auchincloss AH, Michael YL (2015). Impact of policy and built environment changes on obesity-related outcomes: a systematic review of naturally occurring experiments. Obes Rev.

[24] McKinnon RA, Siddiqi SM, Chaloupka FJ, Mancino L, Prasad K (2015). Obesity-related policy/environmental interventions: a systematic review of economic analyses. Am J Prev Med.

[25] Niebylski ML, Redburn KA, Duhaney T, Campbell NR (2015). Healthy food subsidies and unhealthy food taxation: a systematic review of the evidence. Nutrition.

[26] Robertson-Wilson JE, Dargavel MD, Bryden PJ, Giles-Corti B (2012). Physical activity policies and legislation in schools: a systematic review. Am J Prev Med.

[27] Schultz DJ, Byker Shanks C, Houghtaling B (2015). The impact of the 2009 Special Supplemental Nutrition Program for Women, Infants, and Children food package revisions on participants: a systematic review. J Acad Nutr Diet.

[28] Thow AM, Downs S, Jan S (2014). A systematic review of the effectiveness of food taxes and subsidies to improve diets: understanding the recent evidence. Nutr Rev.

[29] Umstattd Meyer MR, Perry CK, Sumrall JC, Patterson MS, Walsh SM, Clendennen SC (2016). Physical activity-related policy and environmental strategies to prevent obesity in rural communities: a systematic review of the literature, 2002-2013. Prev Chronic Dis.

[30] Wharton CM, Long M, Schwartz MB (2008). Changing nutrition standards in schools: the emerging impact on school revenue. J Sch Health.

[31] Williams AJ, Henley WE, Williams CA, Hurst AJ, Logan S, Wyatt KM (2013). Systematic review and meta-analysis of the association between childhood overweight and obesity and primary school diet and physical activity policies. Int J Behav Nutr Phys Act.

[32] Giustini D, Boulos MN (2013). Google Scholar is not enough to be used alone for systematic reviews. Online J Public Health Inform.

[33] Bramer WM, Giustini D, Kramer BM, Anderson PF (2013). The comparative recall of Google Scholar versus PubMed in identical searches for biomedical systematic reviews: a review of searches used in systematic reviews. Syst Rev..

[34] Boeker M, Vach W, Motschall E (2013). Google Scholar as replacement for systematic literature searches: good relative recall and precision are not enough. BMC Med Res Methodol.

[35] Haddaway NR, Collins AM, Coughlin D, Kirk S (2015). The role of Google Scholar in evidence reviews and its applicability to grey literature searching. PLoS One.

[36] Shultz M (2007). Comparing test searches in PubMed and Google Scholar. J Med Libr Assoc.

[37] Mahood Q, Van Eerd D, Irvin E (2014). Searching for grey literature for systematic reviews: challenges and benefits. Res Syn Meth.

[38] Adams J, Hillier-Brown FC, Moore HJ, Lake AA, Araujo-Soares V, White M, Summerbell C (2016). Searching and synthesising “grey literature” and “grey information” in public health: critical reflections on three case studies. Syst Rev..

[39] Godin K, Stapleton J, Kirkpatrick SI, Hanning RM, Leatherdale ST (2015). Applying systematic review search methods to the grey literature: a case study examining guidelines for school-based breakfast programs in Canada. Syst Rev..

[40] Stansfield C, Dickson K, Bangpan M (2016). Exploring issues in the conduct of web searching and other online sources for systematic reviews: how can we be systematic?. Syst Rev.

[41] Eden J, Levit L, Berg A, Morton S (2011). Finding what works in health care: standards for systematic reviews.

[42] Joanna Briggs Institute. Joanna Briggs Institute reviewers’ manual: 2014 edition. Adelaide: Joanna Briggs Institute; 2014. http://joannabriggs.org/assets/docs/sumari/reviewersmanual-2014.pdf. Accessed 21 Apr 2017

[43] U.S. National Library of Medicine. List of all journals cited in PubMed. 27 Dec 2016. https://www.nlm.nih.gov/bsd/serfile_addedinfo.html. Accessed 20 Apr 2017.

[44] Keeling JW, Turner AM, Allen EE, Rowe SA, Merrill JA, Liddy ED, Turtle HR. Development and evaluation of a prototype search engine to meet public health information needs. In AMIA Annual Symposium Proceedings: 2011; American Medical Informatics Association. 693. PMC3243142 22195125

