# Supplementary material for: Information sources for obesity prevention policy research: a review of systematic reviews
Source: Syst Rev. 2017 Aug 8;6:156. doi: 10.1186/s13643-017-0543-2 (PMC5549286; doi:10.1186/s13643-017-0543-2)
Supplement: Supplementary file 1 — Search strategy to identify systematic reviews in obesity prevention policy. This file contains the search terms used by the authors to search the databases PubMed, Public Affairs Information Service (PAIS), Worldwide Political Science Abstracts, Scopus, and Web of Science. (PDF 253 kb) [file 13643_2017_543_MOESM1_ESM.pdf]

Additional file 1. Search strategy to identify systematic reviews in obesity prevention policy

PubMed:
(policy[ti] OR policies[ti] OR legislat*[ti] OR tax*[ti] OR subsid*[ti] OR "health policy"[majr]) AND (food[ti] OR obesity[ti] OR obese[ti] OR overweight[ti] OR "physical activity"[ti] OR "physical education"[ti] OR exercise[ti] OR nutrition[ti] OR diet[ti] OR dietary[ti] OR "sugar-sweetened beverages"[ti] OR "sugar-sweetened beverage"[ti] OR "competitive food"[ti] OR "competitive beverages"[ti]) AND (systematic[ti] OR meta[ti] OR systematic[sb])

PAIS and Worldwide Political Science Abstracts:
(ti(policy OR policies OR legislation* OR law* OR tax* OR subsid*) OR su("health policy")) AND ((obes* OR overweight OR "physical activity" OR "physical education" OR exercise OR nutrition OR diet* OR "sugar-sweetened beverages" OR "sugar-sweetened beverage" OR "competitive food" OR "competitive beverage" OR "competitive beverages" OR food) AND ti(systematic OR meta* OR review))

Scopus:
((TITLE ((policy OR policies OR legislation* OR law* OR tax* OR subsid*)) OR TITLE-ABS-KEY (health policy)) AND TITLE-ABS-KEY ((obesity OR obese OR overweight OR physical activity OR physical education OR exercise OR nutrition OR diet OR dietary OR sugarsweetened beverages OR sugar-sweetened beverage OR competitive food OR competitive beverage OR competitive beverages OR food))) AND (systematic OR meta* OR review)

Web of Science:
((TI=(policy OR policies OR legislation* OR law* OR tax* OR subsid*) OR TS=("health policy")) AND TS=(obes* OR overweight OR "physical activity" OR "physical education" OR exercise OR nutrition OR diet* OR "sugar-sweetened beverages" OR "sugar-sweetened beverage" OR "competitive food" OR "competitive beverage" OR "competitive beverages" OR food) AND ti=(systematic OR meta* OR "rapid review" OR scoping OR umbrella))
