# Supplementary material for: Information sources for obesity prevention policy research: a review of systematic reviews
Source: Syst Rev. 2017 Aug 8;6:156. doi: 10.1186/s13643-017-0543-2 (PMC5549286; doi:10.1186/s13643-017-0543-2)
Supplement: Supplementary file 2 — Systematic reviews included in analysis. This table lists and describes the 21 systematic reviews analyzed in this paper, including metadata (review authors, title, year, journal), review objectives, and major findings. (PDF 110 kb) [file 13643_2017_543_MOESM2_ESM.pdf]

Systematic reviews included in analysis

Authors (year)	Title	Journal	Primary objective	Primary outcome variable(s)	Findings	Geography	Timeline (dates searched)
Alagiyawanna et al. (2015)	Studying the consumption and health outcomes of fiscal interventions (taxes and subsidies) on food and beverages in countries of different income classifications: a systematic review	BMC Public Health	'to study the behavioural and health outcomes of implemented food and beverage FIs in the form of taxes and subsidies in countries of different income classifications'	health and behavioral: '(i) anthropometric measurements, e.g., body mass index (BMI) […], (ii) nutrient intakes (iii) any health outcomes related to diet[…], (iv) pregnancy-related outcomes […]'	'Evidence suggests that [fiscal interventions] on foods can influence consumption of taxed and subsidized foods and consequently have the potential to improve health.'	USA, Australia, Brazil, China, Egypt, Ireland, South Africa	Earliest records to July 2013
Cabrera Escobar et al. (2013)	Evidence that a tax on sugar sweetened beverages reduced the obesity rate: a meta-analysis	BMC Public Health	'to evaluate... the literature on SSB taxes or price increases, and their potential impact on consumption levels, obesity, overweight and body mass index (BMI). The possibility of switching to alternative drinks is also considered.'	'consumption of SSBs, consumption of other drinks, or weight loss, obesity or BMI'	'All showed negative own-price elasticity, which means that higher prices are associated with a lower demand for SSBs.'<br>'Six articles from the USA showed that a higher price could also lead to a decrease in BMI, and decrease the prevalence of overweight and obesity.'	USA, Brazil, France, Mexico	January 2000 to January 2013
Calancie et al. (2015)	Nutrition-related policy and environmental strategies to prevent obesity in rural communities: a systematic review of the literature, 2002-2013	Preventing Chronic Disease	'to synthesize available evidence on the adaptation, implementation, and effectiveness of policy and environmental obesity-prevention strategies in rural settings.'	healthier food environments and policies, consumer behaviors (intentions to consume healthy foods, dietary knowledge, self-efficacy, fruit and vegetable purchasing, reducing intake of sugar-sweetened	'Though results were mixed, interventions tended to improve participants' intentions to consume healthier foods, dietary knowledge, and self-efficacy related to healthy food acquisition and consumption. Also, interventions positively influenced the following behaviors: fruit and vegetable purchasing, reducing intake of sugar-sweetened beverage, and reducing dietary fat intake.'	USA, Canada	January 2002 to June 2013

Authors (year)	Title	Journal	Primary objective	Primary outcome variable(s)	Findings	Geography	Timeline (dates searched)
				beverage, reducing dietary fat intake), weight status			
Cavill et al. (2008)	Economic analyses of transport infrastructure and policies including health effects related to cycling and walking: a systematic review	Transportation Policy	'to review recent approaches to cost-benefit analysis of transport-related physical activity'	cost-benefit ratio, including data on walking/cycling and health effects related to physical activity	'cost-benefit analyses of cycling and walking infrastructure generally produce positive benefit-cost ratios'	USA, England, Finland, The Netherlands, Norway, Sweden	Not available
Chriqui et al. (2014)	Influence of school competitive food and beverage policies on obesity, consumption, and availability: a systematic review	JAMA Pediatrics	'to examine the potential influence that the [USDA interim final rule governing competitive foods and beverages] may have based on peer-reviewed published studies examining the relationship between state laws and/or school district policies and student body mass index (BMI) and weight outcomes, consumption, and availability of [competitive foods and beverages]'	BMI, weight outcomes, consumption, availability of competitive foods and beverages	'In most cases, [competitive food and beverage] policies are associated with changes in consumption and/or availability in the expected direction; however, caution should be exercised, given that nearly all were cross-sectional. The influence of such policies on overall student consumption and BMI and weight outcomes was mixed.'	USA	January 2005 to March 2013
Downs et al. (2013)	The effectiveness of policies for reducing dietary trans fat: a systematic review of the evidence	Bulletin of the World Health Organization	'to systematically review evidence for the effectiveness of policies, including self-regulation, aimed at reducing industrially produced trans fatty acids (TFAs) in food.'	'[trans-fatty acid] levels in food, people's diets, blood or breast milk'	'Overall, the TFA content of food decreased with all types of policy intervention.'<br>'National and local bans were most effective at eliminating TFAs from the food supply, whereas mandatory TFA labelling and voluntary TFA limits had a varying degree of success, which largely depended on food category.'	USA, Brazil, Canada, Costa Rica, Denmark, Netherlands, Republic of Korea	1990 to 2012
Freudenberg et al. (2015)	The state of evaluation research on food policies to reduce obesity and diabetes among adults in the United States, 2000-	Preventing Chronic Disease	'to determine whether waiting for evidence on the impact of changes in dietary policy is preferable to weighing the available	'purchasing behavior, consumption behavior, or body weight/BMI'	'Only 2 of the 5 strategies, menu labeling and taxes on unhealthy foods, had 50% or more studies with positive findings in at least 2 of 3 assessment categories.'	USA	January 2000 to December 2011

Authors (year)	Title	Journal	Primary objective	Primary outcome variable(s)	Findings	Geography	Timeline (dates searched)
	2011		evidence to inform current policy’		‘[M]ost dietary policy strategies lack sufficient evidence to determine whether or not they are effective across settings and populations.’		
Haack & Byker (2014)	Recent population adherence to and knowledge of United States federal nutrition guides, 1992-2013: a systematic review	Nutrition Reviews	‘to summarize population adherence to and knowledge of these corresponding US nutrition guides issued since 1992, including the Food Guide Pyramid, MyPyramid, and MyPlate’	adherence (dietary intake), knowledge of guidelines	‘Across studies, adherence to nutrition guides was low, with participants consuming inadequate levels of fruit, vegetables, and dairy in particular. Knowledge of nutrition guides increased over time since publication and decreased with age of the participants. An association between knowledge of and adherence to nutrition guides was not found.’	USA	January 1992 to October 2013
Jaime & Lock (2009)	Do school based food and nutrition policies improve diet and reduce obesity?	Preventive Medicine	‘to review the effectiveness of school food and nutrition policies world wide in improving the school food environment, student's dietary intake, and decreasing overweight and obesity’	‘menu composition, availability or sales of food and beverages at school, and student’s dietary intake or body mass index (BMI)’	‘Most evidence of effectiveness was found for the impact of both nutrition guidelines and price interventions on intake and availability of food and drinks, with less conclusive research on product regulation.’	USA, Belgium, Denmark, Norway, The Netherlands, Spain, UK	Earliest records to November 2007
Maniadakis et al. (2013)	A systematic review of the effectiveness of taxes on nonalcoholic beverages and high-in-fat foods as a means to prevent obesity trends	ClinicoEconomics and Outcomes Research	‘to assess the possible effects of taxation policies by identifying and analyzing all studies which investigate the impact of price increases on consumption, caloric intake, or weight outcomes’	‘consumption, caloric intake, or weight’	‘Price increase may lead to a reduction in consumption of the targeted products, but the subsequent effect on caloric intake may be much smaller. Only a limited number of the identified studies reported weight outcomes, most of which are either insignificant or very small in magnitude to make any improvement in public health.’	USA, Australia, Brazil, Denmark, France, Germany, Italy, Mexico, The Netherlands, Norway, Singapore, Taiwan, UK	1990 to February 2013

Authors (year)	Title	Journal	Primary objective	Primary outcome variable(s)	Findings	Geography	Timeline (dates searched)
Mayne et al. (2015)	Impact of policy and built environment changes on obesity-related outcomes: a systematic review of naturally occurring experiments	Obesity Reviews	to examine 'the use of natural- or quasi-experiments to evaluate the efficacy of policy and built environment changes on obesity-related outcomes'	obesity/weight/ BMI, physical activity, nutrition/diet	'[S]ome policy and built environmental interventions, especially active transportation infrastructure improvements, bans or restriction on unhealthy foods, and altering purchase/payment rules for low-income food vouchers, can increase certain types of physical activity and improve diet. It is not clear, however, whether these changes result in reduced obesity[...]'	USA, Australia, Canada, Chile, New Zealand, UK	January 2005 to January 2014
McKinnon et al. (2016)	Obesity-related policy/environmental interventions: a systematic review of economic analyses	American Journal of Preventive Medicine	to summarize 'the cost-benefit or cost-effectiveness studies of obesity-related policy/environmental interventions for youth and the general population'	cost-benefit, cost-effectiveness	There was 'relative paucity of studies located conducting cost-benefit or cost-effectiveness assessments of obesity-related policy and environmental interventions in the published literature.'<br>'The vast majority of included studies reported beneficial economic outcomes of the interventions.'	USA, Australia, China, Colombia, Mexico, The Netherlands, New Zealand, UK	January 2002 to January 2014
Niebylski et al. (2015)	Healthy food subsidies and unhealthy food taxation: a systematic review of the evidence	Nutrition	'to evaluate the evidence base to assess the effect of healthy food/beverage subsidies and unhealthy food/ beverage taxation'	blood pressure, BMI, blood lipids or glucose; healthy fruit and vegetable purchases; increased consumption of healthier foods/ beverages, reduced consumption of unhealthy foods/ beverages	'[T]here was consistent evidence that taxation and subsidy intervention influenced dietary behaviors.'	USA, Australia, Canada, New Zealand, Western Europe (various)	June 2003 to November 2013

Authors (year)	Title	Journal	Primary objective	Primary outcome variable(s)	Findings	Geography	Timeline (dates searched)
Powell et al. (2013)	Assessing the potential effectiveness of food and beverage taxes and subsidies for improving public health: a systematic review of prices, demand and body weight outcomes	Obesity Reviews	to review 'recent U.S. studies on [1] the price elasticity of demand for sugar-sweetened beverages (SSBs), fast food and fruits and vegetables as well as [2] the direct associations of prices/taxes with body weight outcomes'	[1] price elasticity of demand<br>[2] body weight	[1] '[T]he price elasticity of demand for SSBs, fast food, fruits and vegetables was estimated to be −1.21, −0.52, −0.49 and −0.48, respectively.'<br>[2] '[Soda taxes] showed minimal impacts on weight; however, [studies] were based on existing state-level sales taxes that were relatively low. Higher fast-food prices were associated with lower weight outcomes particularly among adolescents [....] Lower fruit and vegetable prices were generally found to be associated with lower body weight outcomes among both low-income children and adults [....]'	USA	January 2007 to March 2012
Robertson-Wilson et al. (2012)	Physical activity policies and legislation in schools: a systematic review	American Journal of Preventive Medicine	to 'examine evaluation of school-based physical activity policies [at state and federal levels] for youth over the past 10 years'	implementation, student physical activity	'[S]uch policies can affect health outcomes, specifically by increasing levels of physical activity.'	USA	January 2000 to January 2011
Schultz et al. (2015)	The impact of the 2009 Special Supplemental Nutrition Program for Women, Infants, and Children food package revisions on participants: a systematic review	Journal of the Academy of Nutrition and Dietetics	to assess 'the influence that the 2009 WIC food package revisions have had on dietary intake, healthy food and beverage availability, and breastfeeding participation'	'dietary intake, healthy food and beverage availability, and breastfeeding participation'	The literature indicated 'an improved dietary intake and an increase in the availability of healthier foods and beverages in authorized WIC stores. The revised food package was also associated with improved dietary intake of WIC participants. Mixed results were demonstrated in regard to improved breastfeeding outcomes.'	USA	2009 to April 2014
Thow et al. (2014)	A systematic review of the effectiveness of food taxes and subsidies to improve diets: understanding the recent evidence	Nutrition Reviews	to evaluate 'new evidence published between January 2009 and March 2012 for the effect of food taxes and subsidies on consumption'	overall diet or calorie intake, individual food consumption	'Taxes and subsidies are likely to be an effective intervention to improve consumption patterns associated with obesity and chronic disease, with evidence showing a consistent effect on	USA, Australia, Brazil, New Zealand, Europe (various), UK	January 2009 to March 2012

Authors (year)	Title	Journal	Primary objective	Primary outcome variable(s)	Findings	Geography	Timeline (dates searched)
					consumption across a range of tax rates emerging.’		
Umstattd Meyer et al. (2016)	Physical activity-related policy and environmental strategies to prevent obesity in rural communities: a systematic review of the literature, 2002-2013	Preventing Chronic Disease	to ‘synthesize evidence on the implementation, relevance, and effectiveness of physical activity-related policy and environmental strategies for obesity prevention in rural communities’	implementation success	‘Seven of the 12 [Common Community measures for Obesity Prevention (COCOMO)] physical activity-related strategies were successfully implemented in 2 or more studies, suggesting that these 7 strategies are relevant in rural communities and the other 5 might be less applicable in rural communities.’	USA, Canada	2002 to 2013
Wharton et al. (2008)	Changing nutrition standards in schools: the emerging impact on school revenue	Journal of School Health	‘to examine the available data on what happens financially when strong nutrition standards are applied to foods sold in schools and across districts’	National School Lunch Program participation, food service or school revenue	‘Thus far, few data exist to substantiate the concern that changes in nutrition standards in schools lead to a loss in total revenue. An interesting phenomenon of increased participation in the National School Lunch Program was noted in a number of reports and might play a role in buffering financial losses.’	USA	1997 to date of search
Williams et al. (2013)	Systematic review and meta-analysis of the association between childhood overweight and obesity and primary school diet and physical activity policies	International Journal of Behavioral Nutrition and Physical Activity	‘to evaluate the effects of policies related to diet and physical activity in schools, either alone, or as part of an intervention programme on the weight status of children aged 4 to 11 years’	BMI	‘[W]hen implemented alone, school diet and physical activity related policies appear insufficient to prevent or treat overweight or obesity in children, however, they do appear to have an effect when developed and implemented as part of a more extensive intervention programme.’	USA, Australia, Canada, Italy, Mexico, UK	Earliest records to June 2011
